# Eyes Wide Open, Blinders Attached

**DOI:** 10.4269/ajtmh.18-0126

**Published:** 2018-07

**Authors:** Claire Panosian Dunavan

**Affiliations:** Division of Infectious Diseases, Department of Medicine, David Geffen School of Medicine, University of California, Los Angeles, Los Angeles, California

 *“Okay, on the count of three! Duck. Aim for the door. Then run like hell to the van,” hissed my grim-faced companion, his voice low, his Oxford-cloth shirt darkened with sweat. He’d fought in the Vietnam War. He knew the drill. “The pilot’s on board and the plane is ready to go.”*

 *“By now this place must be crawling with insurgents,” he added under his breath, like a hero in a bad Hollywood film. “If we don’t leave now, we’ll be trapped for sure!”*

 *In the final moments before fleeing the hospital on the remote Pacific atoll—its parrot-green lawn filled with sad, disaffected patients—the following thought flashed: How the heck did I get into this mess in the first place?*

 *The truth is, like many global health advocates past and present, I entered with eyes wide open and blinders firmly attached. In other words, I was sadly unprepared for the possible pitfalls and risks of the “good-will mission” I so casually joined. But I’m getting ahead of my story.*

A month earlier, a long-time friend and fellow tropical medicine doctor called me on the phone. “Are you up for a 2-week trip to the Philippines?” Jim asked. “I’d go myself, but my schedule is packed.”

The year was 1987, and, thanks to a brave, bloodless coup, Ferdinand Marcos’s dictatorship had recently toppled. Soon, two West Coast nonprofits gathered 15 tons of medical supplies, drugs, and equipment for the newly freed state. Then China Airlines offered transport, not only for the booty but also for a delegation. With the Hospital Council of Southern California acting as broker, 30 Los Angeles–area doctors and administrators would now accompany the goods, tour the Philippines, and forge new, trans-Pacific bonds—or so it was hoped.

As the grand experiment neared, only one thing was missing: an expert on the tropical ills and subpar health care the ambassadors would inevitably encounter. It did not take me long to say yes.

Three days before takeoff, I met my new companions and gave an initial briefing, then went home and started to pack.

Our first week in Manila gave us plenty to ponder. From our historic hotel facing the palm-lined promenades of Luneta Park and the fortress of Corregidor, we ricocheted from meetings at the Ministry of Health to modern hospitals with gleaming coronary care units to ramshackle clinics with empty pharmacy shelves and crudely lettered signs warning of possible shakedowns by medical imposters. Following a visit to Ferdinand Marcos’s private dialysis suite and his wife’s 2,000-pair collection of shoes, we toured Manila’s infamous Smoky Mountain, a giant landfill whose septic air mingled scents of lung-scorching chemicals with raw, flowing sewage. In a seaside shantytown, we met laughing children in bright, tattered clothes and then, from the doorway of a church, witnessed sad-eyed youngsters huddled around a toddler-sized coffin. Death was clearly no stranger to them.

During the second week, we broke into teams and left Luzon to visit a few of the Philippines’ less familiar islands. On Negros Occidental, skeletal bodies in cribs in a decrepit hospital caused some of our party to gasp. No one in Manila had mentioned the island’s recent famine. In another rustic sickbay on Negros, we met a dying woman with twig-thin arms, grossly swollen legs, and a massive belly who had never before seen a doctor. What shocked me even more was learning she would soon undergo an operation she could not possibly survive.

And so the days passed, a blur of welcoming smiles mixed with every shade of heartbreak. After touching down in the Visayan Island of Samar, however, I also sensed tension. For the first time, we were quizzed about our cargo. “Why didn’t you bring the supplies here?” was the almost accusatory question of a mayor at a morning buffet. Her tone triggered a memory. I did recall some pretty odd looks back in Manila when the island’s name was mentioned. What did I not know about Samar? Were there special facts others should have shared or pointed questions I should have asked before we left the capital?

By now, my little tribe was down to four Americans—two hospital administrators, a child psychiatrist, and myself—plus an amiable rookie from the Ministry of Health. As the mayor’s question sank in, our American bonhomie flagged. How obvious that she would ask it, it suddenly dawned on all of us, and how ridiculous that we could not answer. But, if truth be told, none of us had a clue about the ultimate plan for the planeload of “stuff” the two California nonprofits had piggybacked on our trip.

“How did you learn about it?” I spluttered at last.

“From articles published in Manila. Since then, we have broadcast the news on local radio.”

“As a matter of fact,” the mayor added with an offhand shrug. “Right now, several hundred peasants from the mountains are camped on our hospital lawn, eagerly awaiting treatments and operations by American medical teams. After lunch, you’ll meet them.”

Local people had traveled from villages—camped overnight—and were now jostling for consults, drugs, and procedures? This was never part of the plan! As the sole clinician in our group and its designated “expert,” I felt a sudden, overwhelming desire to be anywhere on Earth other than Samar.

Still, we could not ignore the misunderstanding or the risk that tensions might mount. And so, after a quick, whispered powwow, I and our alpha male—the former Vietnam sergeant-turned hospital CEO—weakly explained that the supplies were still in customs in Manila. Then we reached for our wallets and donated money. In the same breath, we promised to lobby on behalf of Samar as soon as we returned to Manila. At first, these responses seemed to satisfy the mayor. With momentary relief, I forked a few more bites of *pancit* and mango tart, albeit without much zest.

Then we drove to the hospital, craned our heads, and slowly took in the restive sea of far more people than advertised, perhaps a thousand or more. Walking down a narrow concrete path lined with would-be patients with tumorous goiters, blackened feet, palsied children, and worse—their expressions at once hopeful, poignant, and beseeching—my heart lurched in my chest. Scattered among the crowd, I also spied men in crisply ironed shirts who looked a lot like organizers—or were they paramilitary? I now wonder. Finally crossing a threshold, we took refuge in a room with iron grille windows and faced our worst fears. If anger flared, what then? Would we need to hand over more cash? Might we even be taken hostage? With hundreds of watery miles between us and Manila, one thing was clear. We were on our own.

That’s when someone finally acted. Our junior colleague from the Ministry of Health placed an urgent call to the airfield. The former sergeant took over from there.

In 1999, my husband and I returned to the Philippines to shoot “Hepatitis B—The Global Challenge,” an overseas television special which later aired in multiple Asian countries. Although we did not stop in Samar, I always hoped our program reached the island. A token reparation, I know. Looking back on my 1987 visit, there are many things I would do differently today, starting with a deeper dive into local politics plus a smarter take on our trip’s ultimate goal and chance of success. Never since 1987 have I traveled with a planeload of “unearmarked” medical cargo nor have I joined a group of overseas ambassadors with such varied agendas ranging from genuine altruism to a quest for adventure to a keen instinct for international public relations.

On the other hand, of one thing I remain sure: despite my blinders going in, the threat on Samar was real. I know this because—after returning to Los Angeles—I finally learned some cold, hard facts about the Philippines, a country whose people I continue to love and respect. Before, during, and after 1987, Samar has remained the principal stronghold of the Philippines’ homegrown Maoist insurgency called the New Peoples’ Army (NPA) and an ongoing site of erratic, often deadly violence.

All this history raises troubling questions, even 30 years later. In planning our itinerary, what did our leaders know of Samar, why did they ask us to go there, and how did they later explain our hapless retreat? Did local officials consciously misstate our mission in their radio broadcast—or was it all just a giant mix-up? Did our failure to connect with desperately needy patients ultimately help the NPA, making us pawns in their game? Finally, after my macho companion instructed us to duck, cover, and run instead of weathering the storm and meeting the local Catholic bishop, did donated drugs and supplies ever reach the island?

Somehow, I think not.

